# Adoptive Cell Therapies for Glioblastoma

**DOI:** 10.3389/fonc.2013.00275

**Published:** 2013-11-11

**Authors:** Kevin Bielamowicz, Shumaila Khawja, Nabil Ahmed

**Affiliations:** ^1^Center for Cell and Gene Therapy, Baylor College of Medicine, Houston, TX, USA; ^2^Texas Children’s Cancer Center, Baylor College of Medicine, Houston, TX, USA; ^3^Department of Pediatrics, Baylor College of Medicine, Houston, TX, USA; ^4^Baylor College of Medicine, Houston, TX, USA

**Keywords:** GBM, immunotherapy, cell therapies

## Abstract

Glioblastoma (GBM) is the most common and most aggressive primary brain malignancy and, as it stands, is virtually incurable. With the current standard of care, maximum feasible surgical resection followed by radical radiotherapy and adjuvant temozolomide, survival rates are at a median of 14.6 months from diagnosis in molecularly unselected patients (1). Collectively, the current knowledge suggests that the continued tumor growth and survival is in part due to failure to mount an effective immune response. While this tolerance is subtended by the tumor being utterly “self,” it is to a great extent due to local and systemic immune compromise mediated by the tumor. Different cell modalities including lymphokine-activated killer cells, natural killer cells, cytotoxic T lymphocytes, and transgenic chimeric antigen receptor or αβ T cell receptor grafted T cells are being explored to recover and or redirect the specificity of the cellular arm of the immune system toward the tumor complex. Promising phase I/II trials of such modalities have shown early indications of potential efficacy while maintaining a favorable toxicity profile. Efficacy will need to be formally tested in phase II/III clinical trials. Given the high morbidity and mortality of GBM, it is imperative to further investigate and possibly integrate such novel cell-based therapies into the current standards-of-care and herein we collectively assess and critique the state-of-the-knowledge pertaining to these efforts.

## Introduction

Immunotherapy refers to manipulation of a patient’s immune system to treat disease (2). Tumor-associated antigens (TAAs) comprise unique tumor-specific or tumor-associated molecules that are undetectable or modestly expressed on normal tissues but are more avidly represented on tumor cells and or their microenvironment. TAAs could be immunogenic because they are expressed at higher than normal levels for the developmental stage, or they contain a novel peptide sequence generated by gene mutation or rearrangement, and thus serve as attractive targets for immune based-cellular therapies (3).

Active and passive immunotherapies are two different strategies used to generate anti-tumor activity. Active immunotherapy has generally employed specific tumor vaccines and non-specific immune stimulants. The basis of tumor vaccine therapies is that administration of tumor-specific or TAAs in the context of immune costimulation induces tumor-specific immunity, resulting in anti-tumor effects. Cancer vaccines aim to induce a response predominated by cytotoxic T lymphocytes (CTLs) that are able to recognize such endogenous antigens (4). This approach can be especially effective in cases in which the tumor expresses an antigen but fails to activate the immune system. To elicit CTL responses, the molecules must be expressed in professional antigen presenting cells (APCs), or the tumor cell itself must be modified to express APC characteristics and function (5).

Passive immunotherapy is also referred to as adoptive immunotherapy. Adoptive immunotherapy involves directly transferring effector immune cells to a host in order to induce anti-tumor activity. Non-specific effector cells such as natural killer (NK) cells and lymphokine-activated killer (LAK) cells are innately reactive as they recognize cell surface abnormalities, such as low expression of major histocompatibility complex (MHC) class I molecules or carbohydrate abnormalities. T cells recognize foreign peptides presented on the cell surface by MHC molecules (6). While T cells specific for tumor antigens can be identified within the tumor tissues or elsewhere, most are present at a low frequency, many have receptors with low avidity for the tumor antigens, and some are commonly anergic. One strategy to overcome these limitations is to activate T cells *ex vivo* to circumvent these limitations and to overcome suppressive factors present *in vivo* thus augmenting the anti-tumor activity (7).

This article will describe the rationale for and state of current cell-based therapy strategies that are being used and investigated to treat glioblastoma. The scope of research being conducted is vast, but we will review some benefits and challenges for these approaches. Additionally, future challenges and directions in cell-based therapies for glioblastoma will be discussed. The hope is to present an overview of this novel field as a new adjunct for the current standard treatment of this highly malignant and fatal disease.

## Rationale for Immunotherapy in Glioblastoma

The very poor outcomes for glioblastoma (GBM) using standard-of-care treatment call for novel biologically based interventions. The increasing use of immunotherapeutics stems from the growing body of knowledge of how the immune system interacts with cancer cells and their induced microenvironment. The immune system has both host-protective and tumor-promoting functions, a concept termed cancer immunoediting. This concept describes the immune system’s role in immunosurveillance, maintaining tumor latency, and tumor immune escape mechanisms (8). Tumor cells escape recognition by the immune system by employing a number of strategies including antigen mutation, down regulation and deletion of target antigens, and selective survival of certain antigen negative or positive tumor subpopulations (9, 10). These strategies are particularly relevant to GBM, which is known to be heterogeneous with varying antigen expression profile within single tumors and between patients (11, 12).

Glioblastoma has a number of immunosuppressive properties, and enhancing the host’s immune response against tumor represents a rational approach to reverse this deficiency. Several mechanisms contribute to this immunosuppression. More specifically, GBM tumor cells secrete a myriad of immune-inhibitory molecules such as the transformation growth factor receptor (TGF) β. Increased concentrations of these factors decrease host anti-tumor response and may promote tumor invasion (13). GBM cells are also inefficient in antigen processing, especially in cancer stem cells (CSCs) isolated from this tumor type. This may render GBM cells resistant to the T cell-mediated immune reactions (14).

A subset of GBM cells expressing the surface antigen CD133 exhibit properties of stem cells and the ability to initiate a tumor (15). GBM-associated CSCs have immunosuppressive properties and show resistance to standard therapies. Studies on whether CSCs in solid tumors are more chemo- and or radio-resistant than the bulk population are most advanced for brain cancer. GBM-associated CSCs have immunosuppressive properties and show resistance to standard therapies. CD133+ cells are more resistant to ionizing irradiation than CD133− cells. Expansion in the CD133+ subset was found following irradiation. CD133+ cells preferentially activate the DNA damage checkpoint response more effectively than CD133− cells. CSC population appears to have evolved a more efficient DNA damage repair system than the bulk of the tumor, conferring resistance to radiation treatment (16).

Glioblastoma-associated CSCs participate in tumor-mediated immunosuppression by both secreted and membrane-associated mechanisms and inhibit both innate and adaptive immunity. CD133+ CSCs cells also inhibit T cell proliferation and activation, induce regulatory T cells, and trigger T cell apoptosis (17). These immunosuppressive properties were diminished on altering the differentiation of the cancer-initiating cells (18). Additionally, CSCs recruit monocytes to the tumor microenvironment and contribute to the conversion of monocytes to an immunosuppressive phenotype by a variety of secreted factors. Such macrophages then act in a protumoral manner by enhancing invasiveness, increasing tumor angiogenesis, and potentiating tumor-mediated immunosuppression by a variety of secreted products, while GBM CSCs impede their phagocytic ability (19).

Steroid and radiation therapy for GBM also enhances immunosuppression in these patients (20). As therapy with temozolomide and radiation therapy have now become the standard of care (1), this therapy in the treatment of high-grade gliomas (HGGs) has been associated with lower CD4 counts in some patients. Those with lower CD4 counts were shown to have decreased survival due to earlier progression of their GBM (21).

## Advantages of Cellular Therapeutics

Cell therapy has substantial theoretical and practical advantages over traditional chemotherapy and other immunotherapy strategies. Results from completed phase I/II clinical trials with epidermal growth factor receptor (EGFR) monoclonal antibodies, tumor cell, or dendritic cell (DC) vaccines were encouraging, demonstrating disease stabilization, and prolonged patient survival (22–26). With respect to EGFR monoclonal antibodies, however, one phase I/II trial did not suggest the addition of an anti-EGFR monoclonal antibody had improved efficacy over baseline therapies for progressive disease (27). Mutations in the EGFR receptors on GBM cells can block the action of these therapies in a subset of tumor cells (28).

These trials have highlighted some of the limitations of current approaches. Antibodies – unlike T cells – do not cross the blood brain barrier (BBB), actively migrate through microvascular walls, or penetrate the core of solid tumors to exert their anti-tumor activity. The *in vivo* induction of antigen-specific T cells using antigen loaded DC is often not reproducible, because tumor-specific T cells are either present at very low frequency or are anergized. Tumor cell vaccines contain ubiquitous as well as TAAs and may induce immune responses to normal brain tissue. One strategy to overcome the current limitations of GBM targeted immunotherapies is adoptive T cell transfer in which tumor-specific T cells are prepared *ex vivo* and then transferred into patients (24–26).

In adoptive T cell transfer, it is necessary to identify only a small number of anti-tumor cells with the appropriate properties that can then be expanded to large numbers *ex vivo* for subsequent adoptive transfer. *In vitro* assays can identify the exact populations and effector functions required for cancer regression, which can then be selected for expansion. The cells can be activated in the laboratory free from endogenous inhibitory factors and thus can be induced to exhibit the required anti-tumor effector functions. It is possible to manipulate the host before cell transfer to provide an optimal environment for the transferred cells.

Cell-based therapies provide strategies to overcome these immune effects. Cell-based therapies for the treatment of glioblastoma have evolved from non-specific to more tumor-specific over time (Table 1). Early work introduced NK cells, LAKs, and gamma-delta (γδ) T cells as ways to expand and activate the immune system and tip the balance toward an anti-tumor effector function in the face of a substantially immunosuppressive tumor microenvironment. Other newer approaches use tumor specificity to target tumor-specific antigens using virus specific CTLs and chimeric antigen receptor (CAR) T cells.

**Table 1:** **Modalities of cellular therapies for GBM**.

Cellular product	Description	Mechanism of killing	Advantages	Disadvantages	Reference
Natural killer (NK) cells	Innate immune subset of cytotoxic T cells CD16 and CD56 positive Anti-tumor and antiviral killing	Identify cells lacking MHC-1 for killing Inhibitory signals are not engaged, activating targeted killing	Tumor specimen not necessary for expansion Short expansion time	Non-specific killing Can inhibit virotherapy	Miller et al. (29), Campbell and Hasegawa (30), Dix et al. (33), Miller et al. (35)

Lymphokine-activated killer (LAK) cells	Autologous peripheral lymphocytes activated with IL-2 *in vitro* to become anti-tumor effectors	Unclear	MHC-independent killing	Non-specific killing	Dillman et al. (37)
	Injected with IL-2 when administered to patients	

γδ T cells	Innate effectors that display alternative T cell receptors to αβ T cells	Unclear	MHC-independent killing	Non-specific killing	Bryant et al. (40)

Donor lymphocyte infusion (DLI)	Infusion of donor lymphocytes for anti-tumor effect	Unclear	Graft versus tumor effect	Risk for graft-versus-host disease	Collins et al. (41), Hasskarl et al. (42)

Allogeneic mixed lymphocyte reactive T cells	Lymphocytes from histoincompatible allogeneic donors are mixed with patient lymphocytes and infused	Targeted killing due to sensitization of allogeneic cells to patient MHC	Easy to produce	Risk for alloreactivity	Amrolia et al. (46)

Tumor infiltrating lymphocytes (TILs)	Cytotoxic lymphocytes obtained from tumor site	Through TCR engagement	Tumor-specific killing Cells track effectively to CNS	Cells required from tumor or tumor- draining lymph nodes	Kruse et al. (50), Plautz et al. (53)
	Expanded in presence of IL-2 and original tumor			
	Patient may have preconditioning with radiation or chemotherapy	

Cytotoxic T lymphocytes	Autologous T cells activated and expanded	TCR engagement of antigen in the context of an MHC molecule leads to cytotoxic killing	Targeted killing with specific TCRs	MHC-restricted operation	Dudley et al. (55), Shu et al. (57), Ghazi et al. (65), Ahmed (66)
	Viral or tumor antigens may be targeted		Very effective for virus-driven malignancies	Anergy at low frequency Time to production can be limiting Tregs at tumor site can inhibit function	
			Can be engineered with αβ TCRs or CARs to target tumor antigens		

Chimeric antigen receptor (CAR) T cells	Chimeric molecule Extracellular monoclonal antibody specific for tumor antigen Intracellular T cell signaling domain	Binding of antigen-specific domain initiates T cell-derived signaling which promotes T cell activation and killing	MHC-independent killing Tumor specificity Shorter time to production than CTLs	Off-target effects can occur Limited survival *in vivo*	Eshhar et al. (72, 73)

## Cellular Therapeutic Entities for Glioblastoma

### Natural killer cells

Natural killer cells are a subtype of cytotoxic lymphocytes that participate in the innate immune system. NK cells do not express T cell receptors, T cell marker CD3, or surface immunoglobulin B-cell receptors, but they usually express surface markers CD16 and CD56. They have both anti-tumor and antiviral killing capabilities (29). They kill in an human leukocyte antigen (HLA)-unrestricted manor. Mature NK cells identify normal cells by their expression of inhibitory receptors that recognize MHC-I. When these inhibitory receptors encounter MHC-I on normal cells, they arrest tyrosine kinase-based activation signals. When an NK cell identifies a cell lacking MHC-I, inhibitory receptors are not engaged, and activating signals trigger targeted attack on these cells (30).

Natural killer cells can be activated and expanded *ex vivo* for adoptive immunotherapy. Adoptive immunotherapy with expanded autologous NK cells has resulted in partial responses with minimal toxicity in lymphoma and breast cancer (31). Autologous NK cells have been selectively expanded from PBMCs for patients with high-grade glioma. In the presence of IFN-β as a means to improve NK-mediated cytotoxicity, they were administered intralesionally and IV. 9/16 treatment courses in 9 patients resulted in SD or a measurable response after 4 weeks without severe toxicity (32). Adoptive immunotherapy with NK cells is thought to have several advantages when compared using LAK cells or CTLs. Firstly, tumor specimens are not necessary for autologous NK cell expansion. Additionally, NK cells can be expanded in a relatively shorter time than CTLs and exhibit more robust cytotoxicity compared with LAK cells (32). As with other cellular effectors, the immunosuppressive environment of malignant gliomas suppresses NK activity; T cells from patients with gliomas produce decreased amounts of IL-2 and IFN-γ. These two cytokines are important for the generation of NK activity, and as a result NK function may be impaired in these patients (33).

Natural killer cells have both antiviral and anti-tumor functions, and they may inhibit the efficacy of virotherapy by promoting enhanced tumor cell killing. Upon infection by oncolytic herpes simplex virus, increased expression of certain cell surface proteins can lead to an increase in NK cell activation within the tumor microenvironment. NK cells then kill viral cells, resulting in decreased viral titers and potentially decreasing the efficacy of viral oncolysis (34). Numerous strategies are being investigated to improve NK cell ability to attack cancer cells *in vivo*, one of which is using haploidentical NK cell infusions (35).

### Lymphokine-activated killer cells

*In vitro*, high concentrations of IL-2 can activate a subpopulation of peripheral CD8+ cells to become non-specific tumoricidal effectors; LAK cells. LAK cells can lyse NK cell resistant, fresh, or cultured tumor target cells *in vitro*, including malignant gliomas and other primary brain tumors. LAK cells and IL-2 have been safely administered within the CNS resulting in improved long-term survival in patients with recurrent glioma (36). Significant biologic changes also occurred in these patients including regional eosinophilia and extensive lymphocytic infiltration. In a phase II trial of 33 patients with GBM treated with intralesional autologous LAK cells after initial primary treatment, those with higher doses of LAK cells had longer survival, and overall survival was encouraging compared to controls. Patients on corticosteroids prior to leukapheresis had a lower number of NK cells harvested and lower overall survival (37). LAK cells are also vulnerable to inhibitory molecules expressed by GBM cells (38).

### Gamma-delta T cells

Gamma-delta T cells are a subset of T cells that express alternative, clonally distributed T cell receptors (TCRs) and function as innate effectors and therefore are restricted in TCR diversity. Compared to αβ T cells, they do not target specific peptide antigens and are not constrained by the selectivity and restriction of the MHC. Although γδ T cells absolute counts are decreased and their proliferative capacity is decreased in the setting of GBM, they can be expanded and activated *ex vivo* and have shown the ability to recognize and kill glioma cells *in vitro* while sparing cultured normal astrocytes (39). Expanded and activated γδ T cells can mediate killing of GBM and reduce tumor progression in mouse models (40). However, to date, there have been no human clinical applications to date for treatment of brain tumors.

### Donor lymphocyte infusion and allogenic mixed lymphocyte reactive T cells

Adoptive immunotherapy with donor lymphocyte infusions (DLIs) after hematopoietic stem cell transplant (HSCT) can effectively augment the graft-versus-leukemia (GVL) effect and effectively eliminate residual disease, especially in CML (41). Its GVL effects make DLI currently one of the most effective strategies for patients with recurrent hematological malignancies after allogeneic HSCT (42). Application of DLI is limited by the development of graft-versus-host disease (GVHD), thus DLI is not used routinely used for prophylaxis of relapse after allogeneic HSCT (43). Many strategies have been developed to promote anti-tumor effects of DLIs while decreasing the incidence of GVHD. These include CD8+ cell depletion of DLIs (44), apoptosis regulation (45), and selectively deplete the T cell product of alloreactive cells that express activation markers in response to alloantigen (46). Due to potential and probable risks and limitations application of DLI in the context of GBM has not been effectively explored.

Lymphocytes derived from histoincompatible allogenic human blood donors may also be combined with the patient’s irradiated lymphocytes. A mixed lymphocyte reaction sensitizes the allogeneic donor’s peripheral blood mononuclear cells toward patient’s MHC. Since MHC proteins expressed on patient lymphocytes are also expressed on brain tumor (47) but not on normal brain neurons, astrocytes, or oligodendrocytes (48), the tumor-bearing host MHC offers a means for targeted, selective killing by CTLs sensitized to them.

Additionally, if injected directly into the CNS, alloreactive CTLs may be protected from host immune cells long enough to lyse target cells due to the nature of immune responses in the CNS (49). All these features make this technique a promising strategy for brain tumor therapy. In one study, five patients were infused with intracavitary allogeneic mixed reactive T cells to recurrent gliomas. Two had no evidence of disease and three were alive 28 months after initiation of therapy. Alloreactive CTL treatment was efficacious either when one MHC-disparate donor provided effector CTL several times, or multiple MHC-disparate donors provided effector CTL during the treatment period (50).

### Tumor infiltrating lymphocytes

Tumor infiltrating lymphocytes (TILs) are primarily CTLs which recognize proteolytically cleaved intracellular tumor antigen fragments which have become associated with specific MHC-I antigens on the cell surface (49). Expanded TILs have cytolytic activity against the original tumor and in contrast to LAK cells the killing is MHC class I restricted. They are selectively expanded from either tumor or draining lymph node cells via IL-2, then re-stimulated with irradiated or killed tumor cells to maintain T cell specificity. The complex is recognized by the TCR of specific T lymphocytes TILs are relatively specific in terms of their cytotoxicity to tumors, due to the requirement for the presence of tumor antigen fragments associated with specific classes of MHC-I antigens, and therefore have potential advantages over LAK cells in terms of tumor specificity and cytotoxicity (51). Conditioning the recipient with chemotherapy and/or radiation prior to adoptive transfer of these cells increases the response to adoptive immunotherapy with TILs (52). Other advantages of this strategy include minimal clinical toxicity (in comparison with LAK cells) (53) and that effector function can be easily measured by radioisotope release assays. Indeed, local infusion of autologous TILs and rIL-2 has been done without severe toxicity in patients with recurrent gliomas (51). TILs have been used to treat melanoma metastatic to the brain with very favorable responses. This is clear evidence that TILs effectively track to the CNS (54). Technical issues with producing tumor-specific T cells from brain tumor excision samples currently present a major barrier to conducting clinical trials using TILs in glioma – the minority of biopsy specimens yield satisfactory T cell populations, and the process is labor and time intensive (55).

### Cytotoxic T lymphocytes

Tumor-reactive T cells arise in cancer patients and infiltrate their tumors but are not effective because of tumor-induced functional defects. In general, CTLs represent a more targeted strategy for adoptive immunotherapy. In contrast to NK cells, T cells recognize targets through an antigen-specific TCR and interact with targets only if HLA MHC antigens are also recognized. Developing successful CTL therapies depends on the availability of specific antigens as targets and efficient methods for T cell activation and expansion. Both viral and tumor-specific antigens can be used as targets for CTLs.

Autologous T cells can be activated and expanded with a monoclonal antibody against the TCR to create therapeutically effective T cells from tumor-bearing hosts. These can then be re-infused into the host therapeutically. Lymph nodes draining progressively growing tumors are an optimal source of T cells that are sensitized to specific tumor antigens (56). *Ex vivo* activation of tumor-draining LN cells with anti-CD3 monoclonal antibody or bacterial superantigens induces potent effector function (57). This strategy has been used in patients with newly diagnosed malignant gliomas after surgery and radiation therapy. Several objective clinical responses occurred with 4/12 patients showing partial responses and no long-term toxicity (25).

Since latent viruses are thought to be controlled in immunocompetent hosts by CTLs (58), infusion of T cells that target these viruses can offer effective treatment for virus-driven lymphoproliferation and malignancies in immunocompromised patients. Most extensively, EBV-mediated lymphoproliferative diseases and malignancies have been targeted for adoptive immunotherapy with CTLs. Use of CTLs for EBV-driven post-transplant lymphoproliferative diseases (59), lymphomas (60), and nasopharyngeal carcinomas (61) have been utilized with various degrees of success. A large percentage of GBM have been shown to express the CMV immunodominant proteins pp65 and IE1–72 as well as CMV nucleic acid detected by *in situ* hybridization (62–64). CMV-specific CTLs have thus been expanded *ex vivo* from CMV seropositive GBM patients and recognize and kill CMV-expressing autologous tumor cells (65). This has thus prompted the use of CMV-specific CTLs as a therapeutic modality (66) or as a platform for CAR expression (67). CD133, a stem-like cell marker expressed in the glioma cells that is believed to lead to tumorigenesis in the human brain, is an example of a TAA in glioma cells. CD133+ CTLs have been shown to be cytotoxic to glioma stem cells (68). This antigen has also been targeted by *ex vivo* expansion of CD 133+/DC hybrid cells that stimulate CTLs and have shown to be effective in killing glioma cells (69).

As an alternative to generating tumor antigen-specific T cells for treatment of malignancies using APCs, genetic modification of T cells to express a tumor antigen-specific αβTCRs or CARs has gained substantial interest and produced successes (70, 71). One approach has been to produce T cells with novel receptors by introduction of chimeric receptors that have antibody-based external receptor structures and cytosolic domains that encode signal transduction modules of the TCR (72); CARs. These constructs can function to retarget T cells *in vitro* in an MHC unrestricted manner to attack the tumor while retaining MHC-restricted specificity for the endogenous TCR.

### Chimeric antigen receptor T cells

Tumor-specific T cells can be created by genetically modifying T cells with specificity to express tumor-specific CARs. CARs, which are artificial molecules custom made by fusing an extracellular variable domain derived from a high-affinity monoclonal antibody specific for a TAA of interest to an intracellular signaling domain usually derived from the zeta-signaling chain of the TCR (73). On encountering the specific antigen by the extracellular antibody-derived domain, the T cell-derived signaling domain initiates an intracellular signal that results in T cell activation.

T cells with CARs have many potential advantages over immunotherapies based on monoclonal antibodies or T cells alone. CARs recognize antigens in an HLA-independent manner, and thus overcoming a major limitation of the αβ TCR, which is limited by MHC restriction. CARs are able to circumvent some mechanisms by which tumors avoid MHC-restricted T cell recognition, such as down regulation of MHC molecules (74). CAR-expressing T cells are more likely to eradicate tumor cells than antibodies alone, since they can migrate through microvascular walls, extravasate, and penetrate the core of solid tumors to exert their cytolytic activity, and sequentially kill a multiplicity of target cells (75). They react better to modestly expressed tumor targets because they can recruit additional components of the immune system and amplify the anti-tumor immune response. They broaden the range of antigens recognizable by T cells to include carbohydrate and glycolipid antigens. CARs can reliably generate T cells in a relatively short time for clinical usage, typically 10–15 days (75).

IL-13 receptor alpha 2 (IL13Rα2), human epidermal growth factor receptor 2 (HER2), and Ephrin type A receptor 2 (EphA2) are all glioma-specific antigens that provide targets for CAR-based immunotherapy (76–78). The former is currently being explored in a clinical trial infusion autologous CAR T cells clones into resection cavities of GBM (79). T cells from GBM patients have been genetically engineered to be rendered HER2 specific. These effector cells recognized autologous HER2-positive GBMs including their CD133-positive stem cells *in vitro* and had potent anti-tumor activity against both killed CD133-positive and CD133-negative cells derived from primary HER2-positive GBMs (80). T cells engineered to target IL13Rα2 have also shown tumor recognition and anti-tumor effector function (81). A clinical trial co-targeting HER2 and CMV pp65 using autologous bispecific CAR CTLs is currently underway in our center (67). T cells engineered with CARs against such GBM-specific antigens provide promising immunotherapeutic approaches for the treatment of GBM. Another current phase I/II clinical trial is investigating the safety and effectiveness of autologous CARs targeting EGFRvIII in malignant gliomas (82).

## Miscellaneous; Specific Considerations

### Helper T cells

Many approaches to tumor immunotherapy in GBM have focused on the CD8+ T cell effector functions. This is based on the observation that most tumor cells express some degree of MHC class I but not class II molecules. Tumor models have demonstrated that CD4+ tumor-reactive T cells have been shown to eradicate an MHC class I positive but class II negative tumor in the absence of CD8+ T cells for brain neoplasms, possibly showing the importance of interactions between CD4+ T cells and MHC class II expressing tumor-associated cells (83). Because the mechanism of CD4+ cells role in anti-tumor activity is not well defined, it is not yet clear what type of *in vitro* assay would provide an optimal surrogate marker of an effective immune responses. Currently, there are no clinical applications to date using these cells to enhance immunotherapy for GBM (25).

### Regulatory T cells

The main role of regulatory T cells (Tregs) is suppression of the function of other cells. They express CD4 and CD25 on their cell surface. Expression of the specific molecular marker FOXP3 is increased when Tregs are activated. There is a significant increase in the number of FOXP3-expressing CD4+CD25+ T cells within GBM-infiltrating lymphocytes and in the peripheral blood of patients with GBM (84). Treg depletion inhibits growth of GBM tumors, but its efficacy may depend on tumor burden, as this strategy has been shown to be more effective with smaller tumor burdens earlier in the course of disease (85). Tregs also represent an increased fraction of the remaining CD4 compartment in patients with glioblastoma. This increased Treg fraction correlates with and is sufficient to elicit the characteristic manifestations of impaired patient T cell responsiveness *in vitro* (86). Treg removal eradicates T cell proliferative defects and reverses cytokine shifts, allowing T cells from patients with malignant glioma to function *in vitro* at levels equivalent to those of normal, healthy controls. The collective restoration of these immune functions may allow for enhancement of the antiglioma activity of adoptively transferred T cells (87). This increase in the frequency and fraction of Tregs might play a role in modulation of the immune response against malignant brain tumors. Methods to decrease or eliminate Treg function may improve clinical results for immunotherapy patients with GBM in the future.

## Limitations of Cellular Therapeutics

Several obstacles, some posed by glioma cells or their microenvironment and others intrinsic to the cellular products, limit the clinical efficacy of cellular therapeutics. Antigen presentation can be impaired in malignancy by down regulation of the MHC class I molecules on tumor cells, resulting in an inefficient recognition by CD8+ anti-tumor lytic effectors; and the lack of the MHC class II expression by most human tumors, limiting the simultaneous engagement of CD4+ helper response at the tumor site alongside CD8+ lytic response (88). We now have a better understanding of the CD4+ suppressor/Tregs and their influence on the generation of a productive anti-tumor immune response.

Additionally, tumors expressing tumor antigens and costimulatory molecules may not present the tumor peptides because of the interference with the antigen-processing pathway by tumors (89). The expression of peptide transporter molecules required for peptide loading of MHC class I complexes can be downregulated. Tumors may also inhibit cross-priming by professional APCs by secreting inhibitory cytokines, which downregulates MHC class II molecule expression on macrophages and DCs and prevents their release of inflammatory cytokines (90).

The tumor microenvironment produces many obstacles to cell-based therapies. Indeed, tumor cells can, directly or by influencing the tumor microenvironment to play a protumoral role, manipulate the host’s immune response for tumor-protective effects. T cells in the microenvironment and their impact on tumor growth may depend heavily upon the particular TIL subset. The majority of CD4+ T cells favor tumor progression, while CD8+ T cells favor tumor rejection (91). As mentioned above, Tregs inhibit the effect of T cells to tumor or foreign antigens (92). Cytokines produced by tumors stimulate increased helper and Treg function that decreases natural tumor immunity (93). When these regulatory cells are decreased in number, they increase the anti-tumor effectors T cells (94). Suppressing these cells could enhance natural tumor immunity and the ability to use adoptive strategies, while their up regulation formidable obstacle to their effectiveness.

Cellular products do alternatively pose obstacles, the influence of which is progressively decreasing because of our improved understanding of the influence of culture conditions on the quality and reliability of generating effector cells. Problems such as limited *in vivo* expansion post infusion are being resolved by optimizing the cellular product (for example including enhanced signaling domains in CAR T cells or infusion of more naïve phenotypes of effectors) and or optimizing the receiving host by strategies such as lymphodepletion or coadministration of immuno-stimulatory cytokines.

## Challenges and Future Innovations

The BBB provides challenges in using immunotherapy for the treatment of CNS malignancies. Isolation from systemic circulation prevents delivery of T cells to the site of malignant gliomas in the CNS. This barrier also provides immune privilege that makes utilizing host immune responses in treatment of these malignancies challenging (95). There is evidence that TILs cross the BBB from studies showing success using TILs in the treatment of CNS lymphoma (54) and post-transplant lymphoproliferative disease (96–98). The delivery method of immune cells to the tumor can circumvent the BBB. While many studies utilize direct injection of cells to the tumor site or systemic injections, intranodal, intradermal, and intranasal, injections are other options. Investigative comparison of these delivery strategies should be performed to reach the optimal delivery route for effective GBM cellular therapy modalities. It has been shown that the interaction between brain tumor cells and their associated endothelium triggers T cell migration into the tumor microenvironment. Communication between the tumor cells and endothelium with chemokines triggers this T cell migration (99). These interactions should be explored as potential targets to augment adoptive cell therapies.

Glioma cells are also considered to be poor APCs. They have inadequate phosphorylation and cytoskeletal rearrangements which are required for appropriate APC to T cell contacts and stimulation of an immune response. T cells obtained from patients with gliomas do not make sufficient contact with APCs and consequently are not appropriately stimulated, which may explain why only a limited number of T cells migrate into the tumor sites (33).

Combining cell therapies with other novel treatment strategies could lead to better outcomes for patients. One way this can be done is through epigenetic modifiers. Epigenetics is the study of stable genetic modifications that result in changes in gene expression and function without a corresponding alteration in DNA sequence (100). These modifiers can work separate and in addition to conventional therapies or work to enhance conventional therapies. For example, tumorigenicity of glioma stem cells can be inhibited by inhibiting the JAK/STAT signaling pathway, and finding ways to inhibit this and other pathways could have therapeutic effects (101).

Glioblastoma microenvironment produces immunosuppressive cytokines. Preoperative and postoperative plasma levels of active and latent TGF-β are significantly higher in patients with GBM than in plasma from normal controls (13). TGF-β is a cytokine that can have inhibitory effects on the immune system and proliferation and can allow cancers to become more invasive. TGF-β may therefore contribute to depressing the general immune defense system of glioblastoma patients, which furthermore may support the immune surveillance of the tumor. Selective inhibitors of the TGFbR-I kinase can potentiate radiation responses in glioblastoma cells by coordinately increasing apoptosis and cancer stem-like cells targeting while blocking DNA damage repair, invasion, and angiogenesis (102). Alternatively, using human tumor antigen-specific CTLs engineered to resist the inhibitory effects of tumor-derived TGF-β by using a retrovirus vector in which the dominant negative TGF-β type II receptor was modified and ineffective produced TGF-β-resistant CTLs that had a functional advantage over unmodified CTLs in the presence of TGF-β-secreting EBV positive lymphoma. These CTLs also had enhanced anti-tumor activity (103). The potential value of this approach in cell-based therapies is currently being explored further (104).

Glioblastoma is the epitome of heterogeneity and targeted cell therapies can become ineffective over time as tumors develop antigen escape variants. This could develop because of the high mutation rate in GBM, but might be intrinsic to the tumor cell heterogeneity or induced by selective survival of target negative cells after therapy. Overcoming this mechanism of resistance will be necessary to improve response in patients. Along this line, there are strategies in the development that could offset antigen escape by co-targeting multiple TAA using cellular products grafted with multiple CARs (10) or single CAR molecules that can mediated bispecific activation and targeting of T cells (105).

Phase II clinical trials are needed to determine efficacy of immunotherapies (Table 2). Designing these trials with effective monitoring of immunotherapy and immune system-related endpoints to assess disease response can be challenging. The modified Response Evaluation Criteria in Solid Tumors (RECIST) criteria are a commonly used tool to assess solid tumor disease response to therapeutic agents. In general, it relies on the presence of disease on imaging at the beginning of a treatment, and uses enhancement to gage response to the therapy (106). Since anti-tumor immune responses may be delayed compared to conventional chemotherapy and new or larger enhancing lesions soon after immune therapy may represent immune cell infiltration, immune-related response criteria (irRC) have been developed to incorporate these principles (107). Immunotherapy can also induce serum or local cellular responses, but immune response assays to define biomarkers of immune response often have highly variable and often non-reproducible results (108).

**Table 2:** **Current clinical trials of adoptive cellular therapy for GBM**.

Trial/phase	Cell therapy type	Description	Sponsor/clinicaltrials.gov identifier
Phase II study of intralesional adoptive cellular therapy of GBM with interleukin-2-stimulated lymphocytes	LAK cells	Determine the feasibility, side effects, and toxicity associated with intracranial cellular adoptive immunotherapy comprising aldesleukin-stimulated lymphokine-activated killer cells in patients with GBM	Hoag Memorial Hospital Presbyterian/NCT00331526 (completed)

Phase I study of cellular immunotherapy for recurrent/refractory malignant glioma using intratumoral infusions of an allogeneic genetically modified cytolytic T cells	αβ T cells	To study the safety and feasibility of giving intralesional GRm13Z40-2, an allogeneic CD8(cytotoxic T cell line genetically modified to express the IL-13 zetakine chimeric immunoreceptor and the Hy/TK selection/suicide fusion protein and found to be resistant to corticosteroids together with aldesleukin in treating patients with malignant glioma	City of Hope Medical Center/NCT01082926

Phase I study of recovery from drug-induced lymphopenia using CMV-specific T cell adoptive transfer	Vaccine/CMV-specific cytotoxic lymphocytes	To evaluate if vaccinating patients with newly diagnosed GBMs using CMV-DCs during recovery from therapeutic temozolomide (TMZ)-induced lymphopenia with autologous lymphocyte transfer (ALT) in patients that are seropositive for CMV enhances the T cell response. To evaluate the safety of ALT with CMV pp65-activated T cells in patients with newly diagnosed GBMs during recovery from therapeutic TMZ-induced lymphopenia	Duke University Medical Center/NCT00693095

Phase I study to investigate autologous lymphoid effector cells specific against tumor-cells (ALECSAT) administered to patients with GBM	Autologous T cell infusion	To Assess safety and tolerability for administration of the cell-based immunotherapy ALECSAT to patients with GBM	CytoVac A/S/NCT01588769 (completed)

Phase I/II study administering T cells expressing anti-EGFRvIII CAR	CAR T cells	To evaluate the safety and 6-month progression free survival in patients with malignant gliomas expressing EGFRvIII administered anti-EGFRlll CAR engineered peripheral blood T cells, a non-myeloablative conditioning regimen, and aldesleukin	National Cancer Institute/NCT01454596

Phase I study of cellular immunotherapy for recurrent/refractory malignant glioma using genetically modified autologous T cells	αβ T cells	To assess the feasibility and safety of cellular immunotherapy utilizing *ex vivo* expanded autologous CD8-positive T cell clones genetically modified to express the IL-13 zetakine chimeric immunoreceptor and the Hy/TK selection/suicide fusion protein in patients with recurrent or refractory, high-grade malignant glioma	City of Hope Medical Center/NCT00730613 (completed)

Phase I study of HER2 CAR-expressing CMV-specific cytotoxic T cells in patients with GBM (HERT-GBM)	CAR modified CMV-specific cytotoxic lymphocytes	To evaluate the safety of autologous CMV-specific CTLs genetically modified to express CARs targeting HER2 in patients with HER2-positive GBM who have recurrent or progressive disease after front line therapy	Baylor College of Medicine/NCT01109095

Lastly, while the overall safety of cell therapies has been good, evaluating, and improving the safety of these techniques is essential. Since the immune system is manipulated and activated, there is a concern for pathologic immune activation. Overproduction of proinflammatory cytokines can occur as a result of T cell triggering, a phenomenon known as the cytokine release syndrome (109). Features of this syndrome include high-grade cyclical fevers and hypotension, and it has been reported after infusion of CAR T cells (110). One approach investigated to prevent adverse outcomes is to incorporate a safety or “suicide” gene in the transferred cells. A small molecule is administered in the event of an adverse event activates a suicide-gene product and kills the transduced cell by inducing apoptosis (45).

Cellular therapies are effective, potentially safe options for GBM, they are complex and potentially quite expensive but could improve the dismal outcomes while maintaining a very favorable toxicity profile.

## Conflict of Interest Statement

The Center for Cell and Gene Therapy (CAGT) has research collaboration with Celgene Inc., to develop chimeric antigen receptor (CAR)-based therapeutics, that is administered by Baylor College of Medicine (BCM). Nabil Ahmed has patent applications in the field of T cell and gene-modified T cell therapy for cancer. This work was funded by the Alliance for Cancer Gene Therapy (ACGT, Inc.), Alex’s Lemonade Stand Pediatric Cancer Foundation (ALSF), and the Stand Up to Cancer (SU2C) – St. Baldrick’s Pediatric Cancer Dream Team Grant.
